# A step towards stereotactic navigation during pelvic surgery: 3D nerve topography

**DOI:** 10.1007/s00464-018-6086-3

**Published:** 2018-02-12

**Authors:** A. R. Wijsmuller, C. Giraudeau, J. Leroy, G. J. Kleinrensink, E. Rociu, L. G. Romagnolo, A. G. F. Melani, V. Agnus, M. Diana, L. Soler, B. Dallemagne, J. Marescaux, D. Mutter

**Affiliations:** 1 Department of Surgery, VU University Medical Center, Amsterdam, The Netherlands; 2 IRCAD/ EITS, Department of General, Digestive and Endocrine Surgery, Nouvel Hôpital Civil, University Hospital of Strasbourg, Strasbourg, France; 3 IHU Strasbourg, Institute of Image-Guided Surgery, Strasbourg, France; 4 Department of Digestive Colorectal Minimally Invasive Surgery, Hanoi High Tech and Digestive Center, Saint Paul Hospital, Hanoi, Vietnam; 5 Department of Neurosciences, Erasmus University Medical Center, Rotterdam, The Netherlands; 6 Department of Radiology, Sint Franciscus Gasthuis, Rotterdam, The Netherlands; 7 IRCAD Latin America, Department of Surgery, Barretos Cancer Center, Barretos, Brazil; 8 Americas Medical City, Rio de Janeiro, Brazil; 9 IRCAD Latin America, Rio de Janeiro, Brazil

**Keywords:** Autonomic nervous system, Topography, Medical, Neuronavigation, Anatomy, Magnetic resonance imaging, Hypogastric plexus

## Abstract

**Background:**

Long-term morbidity after multimodal treatment for rectal cancer is suggested to be mainly made up by nerve-injury-related dysfunctions. Stereotactic navigation for rectal surgery was shown to be feasible and will be facilitated by highlighting structures at risk of iatrogenic damage. The aim of this study was to investigate the ability to make a 3D map of the pelvic nerves with magnetic resonance imaging (MRI).

**Methods:**

A systematic review was performed to identify a main positional reference for each pelvic nerve and plexus. The nerves were manually delineated in 20 volunteers who were scanned with a 3-T MRI. The nerve identifiability rate and the likelihood of nerve identification correctness were determined.

**Results:**

The analysis included 61 studies on pelvic nerve anatomy. A main positional reference was defined for each nerve. On MRI, the sacral nerves, the lumbosacral plexus, and the obturator nerve could be identified bilaterally in all volunteers. The sympathetic trunk could be identified in 19 of 20 volunteers bilaterally (95%). The superior hypogastric plexus, the hypogastric nerve, and the inferior hypogastric plexus could be identified bilaterally in 14 (70%), 16 (80%), and 14 (70%) of the 20 volunteers, respectively. The pudendal nerve could be identified in 17 (85%) volunteers on the right side and in 13 (65%) volunteers on the left side. The levator ani nerve could be identified in only a few volunteers. Except for the levator ani nerve, the radiologist and the anatomist agreed that the delineated nerve depicted the correct nerve in 100% of the cases.

**Conclusion:**

Pelvic nerves at risk of injury are usually visible on high-resolution MRI with dedicated scanning protocols. A specific knowledge of their course and its application in stereotactic navigation is suggested to improve quality of life by decreasing the likelihood of nerve injury.

**Electronic supplementary material:**

The online version of this article (10.1007/s00464-018-6086-3) contains supplementary material, which is available to authorized users.

Focus of rectal cancer research has shifted to functional outcomes. Long-term morbidity after multimodal treatment for rectal cancer is suggested to be mainly made up by nerve-injury-related dysfunctions such as urinary, sexual, and anorectal dysfunctions [1–3]. Recently, the performance of stereotactic navigation for minimally invasive transanal rectal surgery was reported [4]. To assess the additional challenges related to stereotactic pelvic navigation compared with surgical navigation in other contexts such as neurosurgery and orthopedic surgery, a study with human male anatomical specimens was performed [5]. It was concluded that accurate stereotactic pelvic surgical navigation should be feasible [5]. Its application is suggested to improve safety and quality of the surgery as has been shown for stereotactic navigation in other contexts [6]. Stereotactic navigation would be more effective when structures at risk of iatrogenic damage, such as the pelvic nerves, are highlighted.

Pelvic nerves consist of sympathetic, parasympathetic, and/or somatic fibers. The pudendal (PN), levator ani (LAN), and obturator nerve (ON) originate from sacral (SNs) and lumbar nerves. In addition, the SNs transfer fibers to the inferior hypogastric plexus (IHP) through the pelvic splanchnic nerves (PSNs), also known as the erigent pillar or nerves. The sympathetic trunk (ST) gives off branches to the SNs and fibers to the IHP through the sacral splanchnic nerves (SSNs). The superior hypogastric plexus (SHP) originates from the inferior mesenteric plexus and follows through in the hypogastric nerves (HNs), which follow through in the IHP. These nerves ultimately innervate the following anatomical structures: bladder, seminal vesicles, prostate, rectum, urethra, pelvic floor, dermis, anal sphincter, the vessels of the lower extremities, adductor muscles as well as corpora cavernosa in men, and uterus and vagina in women. Consequently, damage can result in several types of dysfunction.

In 2014, Bertrand et al. performed pelvic 3-T magnetic resonance imaging (MRI) and dissections in eight adult human anatomic specimens to identify the HN, IHP, PSNs, and cavernous nerves, and they concluded that MRI was suitable to detect autonomous pelvic innervations [7]. The aim of this study was to investigate the ability to make a 3D topographic map of all autonomic and somatic nerves at risk for injury during pelvic visceral surgery with in vivo MRI.

## Methods

The study was made up of two parts. First, a systematic review was performed to identify a main positional reference in relation to anatomical landmarks for each individual autonomic or somatic nerve (i.e., SHP, HNs, IHP, ST, ON, PN, and LAN, except for the lumbosacral plexus and SNs), thereby facilitating their identification on MRI. Second, these nerves were manually delineated in 20 volunteers who were scanned with a 3-T MRI scanner.

### Review

#### Search strategy

This systematic review was performed in accordance with the Preferred Reporting Items for Systematic reviews and Meta-Analysis (PRISMA) Group [8]. A literature search was performed in the following databases: MEDLINE, EMBASE, and Science Citation Index Expanded. The search headings and time span used for each nerve and each database are provided in the Supplementary Table 1. We included English-, French-, German-, and Spanish-language full-text reports. The references of retrieved articles were searched for in order to identify additional studies for inclusion.

Studies in which human anatomic specimens were dissected were included. Descriptions in anatomy books were not searched for, since the anatomical studies on which they were based were included, and mere anatomical descriptions without scientific basis were not included. Studies on fetuses were eligible for inclusion if the fetuses were older than 8 weeks of gestation. After this period, pelvic innervation is mostly mature, stable, and comparable to the one of the adult [9]. For each individual nerve, plexus, or nerve segment, one main reference point was selected in order to facilitate its identification on MRI. During full-text analysis, the most useful reference point was taken. In case another more useful reference point was found in a study analyzed later on, this reference point was used, and the studies which had already been analyzed were analyzed once more for this reference point. If no description was provided regarding the relation of the nerve structure with the reference point in texts or in pictures of the dissections, the study was excluded from any further analysis. Studies which reported on the sympathetic pelvic plexus without differentiating between ST/sympathetic chain and the course of the SHP, HNs, and IHP, were also excluded. In addition, studies in which only the very distal ends of the branches of the PN or the IHP were studied were excluded.

#### Data extraction

Two authors (ARW and CG) independently analyzed the literature. On the basis of title and abstract, a first selection was made, and irrelevant publications were deleted. The remaining records were assessed for eligibility by analyzing the full text after which irrelevant studies were deleted. The remaining studies were included in the quantitative synthesis.

### MRI nerve topography

Secondly, 20 volunteers (10 men/10 women, mean age 36, range 19–58, mean BMI 24.3, range 17.5–31.2) underwent a nonenhanced pelvic MRI exam at the iCube laboratory (IMIS platform, University of Strasbourg) according to the study protocol for which ethics approval was obtained from the Institutional Review Board. Written informed consent was given by all subjects, and they were asked to empty their bladder prior to the MRI examination. No medication was used to reduce bowel motion. The subjects were examined with a 3-T MR system using a 6-channel phased-array flexible coil and a spine coil for signal reception. A dielectric pad was used to prevent standing wave artifact and to limit breathing motion in the upper part of the pelvis. Our strategy was to use and optimize the contrast difference that exists between nerves and pelvic fat in MRI. Consequently, several sequences were tested. A 3D T1-weighted sampling perfection with application of optimized contrasts using different flip-angle evolutions (SPACE) sequence was acquired in all volunteers. A 3D T2-weighted SPACE sequence was also acquired in 85% of volunteers. During the study, two adjustments were made to the protocol in order to optimize the nerve-tracking capability: nonenhanced angiographic images were acquired with a phase-contrast sequence in 95% of volunteers and a 3D proton-density (PD)-weighted SPACE sequence was acquired in 65% of volunteers. The scanning parameters of the different sequences are provided in the Appendix.

The abovementioned nerves were manually delineated (ARW) in consecutive slices using the ITK-SNAP software (Penn Image Computing and Science Laboratory, the Department of Radiology, University of Pennsylvania, USA) for each volunteer [10]. The time required to delineate the nerves was not recorded since this time was not representative for the time spent on delineation alone but also the time spent on recognition of nerves which have not yet been identified on in vivo MRI until now. In addition, an attempt was made to identify the connecting fibers such as PSNs and SSNs. 3D navigation was performed to check the continuity of structures and their anatomical accuracy.

#### Analysis

The identifiability rate for each individual nerve was determined, and the likelihood of nerve identification correctness was verified in three ways. First, it was checked whether the course of the presumed nerve coincided with the positional reference found in our review study. As the second and third controls, an anatomist (GJK) and a radiologist (ER), both specialized in pelvic anatomy, specified their levels of agreement or disagreement regarding the likelihood of correctness for each nerve delineation using a Likert scale (0 = I do not agree that the delineated nerve depicts the correct nerve, 1 = I doubt that the delineated nerve depicts the correct nerve, 2 = I agree that the delineated nerve depicts the correct nerve). The anatomist compared the reconstructed 3D model to the course he expected the nerves would take. The radiologist scored her level of agreement on the same 3-point Likert scale, on the basis of 3D navigation through the sequence used for the delineations. She analyzed the images with and without the delineations of the nerves.

A quality score was given by the radiologist (ER) to the scan of each volunteer with respect to the ability to distinguish the HN on a 5-point Likert scale (1 = poor, 2 = fair, 3 = good, 4 = very good, 5 = excellent). A Chi-square test was used to assess the relationship between gender and this quality score. A one-way analysis of variance (ANOVA) was performed to determine the influence of BMI and age on the Likert score for the quality of the scan.

## Results

### Review of positional references

Separate queries for the search terms provided in Supplementary Table 1 revealed a total of 8168 records. Using the references of the selected articles, 10 more studies were identified. A total of 1986 were discarded as doubles, 6034 were discarded by screening on the basis of title or abstract, and 97 articles were discarded after assessment of the full texts. The PRISM flow diagram (Supplementary Fig. 1) shows the literature screening process for all nerves together. Supplementary Table 2 reports all studies that were included for quantitative synthesis. For each nerve or plexus, a main positional reference was found. These are reported in Table 1.

**Table 1:** The first three columns depict the main positional references identified for each nerve/plexus and records included in the quantitative synthesis. The last columns depict the identifiability and level of agreement with positional references, anatomist, and radiologist

Nerve/ plexus	Records included in quantitative synthesis	Main positional reference	Identifiability (%)^a^	Conform positional reference (%)^b^	Level of agreement anatomist (Likert score 2)	Level of agreement radiologist (Likert score 2)
Lumbosacral plexus	NA	NA	20 (100)	NA	20 (100)	20 (100)
Sacral nerves	NA	NA	20 (100)	NA	20 (100)	20 (100)
Obturator nerve	2 [11, 12]	Appearance lateral to confluence of the internal and external iliac vein	20 (100)	20 (100)	20 (100)	20 (100)
Sympathetic trunk	4 [13–16]	Entrance pelvis from either side of the lumbar spine dorsal from common iliac vein to course medial to sacral foramina	19 (95)	19 (100)	19 (100)	19 (100)
Superior hypogastric plexus	12 [7, 16–26]	Division at or just below the level of the sacral promontory	14 (70)	14 (100)	14 (100)	14 (100)
Hypogastric nerve	12 [7, 16, 19, 21, 25–32]	Course just medial to internal iliac vessels	16 (80)	16 (100)	16 (100)	16 (100)
Inferior hypogastric plexus	17 [7, 16, 21, 25–38]	Ureter crosses, just before entering the bladder, anterior to the IHP	14 (70)	14 (100)	14 (100)	14 (100)
Right pudendal nerve	6 [39–44]	Medial course to the pudendal artery just before passing the ischial spine and leaving the pelvis through the greater sciatic foramen	17 (85)	16 (94)	17 (100)	17 (100)^c^
Left pudendal nerve			13 (65)	11 (85)	13 (100)	13 (100)^c^
Levator ani nerve unilateral	8 [13, 44–50]	A supralevatory course parallel to the course of the PN in a ventral direction	6 (30)^d^	6 (100)	4 (67)^e^	3 (50)^e^

*NA* not applicable

^a^Identifiability is reported for right and left sides together except for the PN and LAN

^b^Percentages are based on the cases in which the nerve/plexus was identified

^c^In one volunteer in whom no PNs were identified, the radiologist identified the PN bilaterally

^d^In 6 volunteers, only on one side, a LAN was identified

^e^In 2 volunteers, the anatomist gave a Likert score 1 since he was in doubt the delineated nerve depicted the LAN. In 3 volunteers, the radiologist gave a Likert score 1 since he was in doubt the delineated nerve depicted the LAN

### Delineations

Two of the twenty volunteers were operated on in the abdomen: one had a subtotal colectomy and the other had a childbirth through a cesarean section. These interventions did not influence pelvic anatomy.

**Fig. 1:**
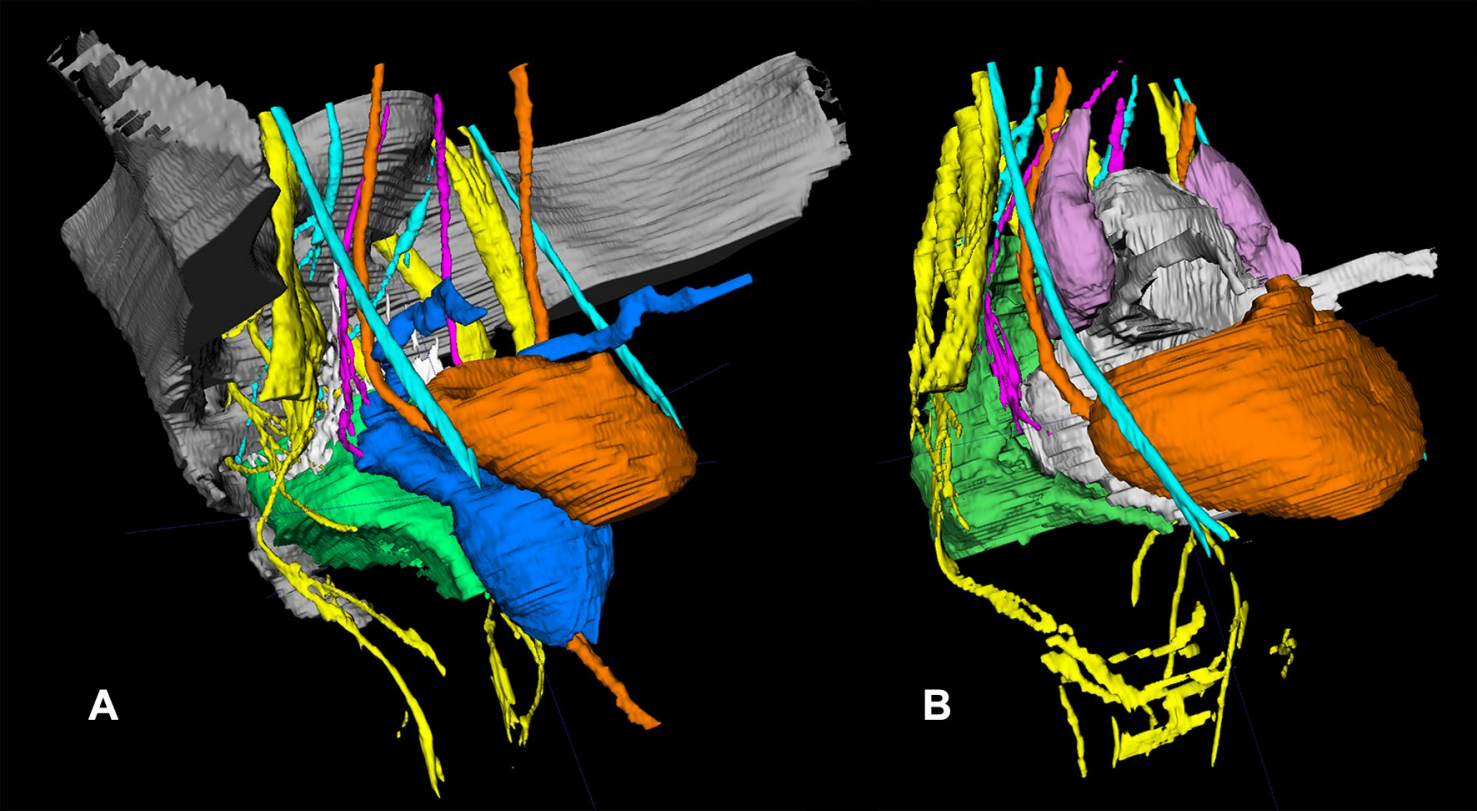
3D MRI pelvic nerve topography showing the anatomical relationships between the pelvic nerves and the pelvic organs in a male (**A**) and in a female volunteer (**B**). A video of such a delineation is available as supplementary material 1. The following pelvic organs and nerves can be distinguished: bladder/ureter and urethra (orange), mesorectum of the low and partially mid-rectum (green), prostate/seminal vesicles/deferent duct (in **A**, blue), sacrum and partially iliac bone (in **A**, gray), uterus/vagina/round ligament (in **B**, gray), ovaria (purple), SNs/lumbosacral plexus/PN/LAN (yellow), ST (turquoise, running ventrally along the sacrum), ON (turquoise, the most ventrally situated nerve), SHP/HN (pink), IHP (in **A**, white/distal side pink; in **B** the pink HN is running through in the IHP). (Color figure online)

**Fig. 2:**
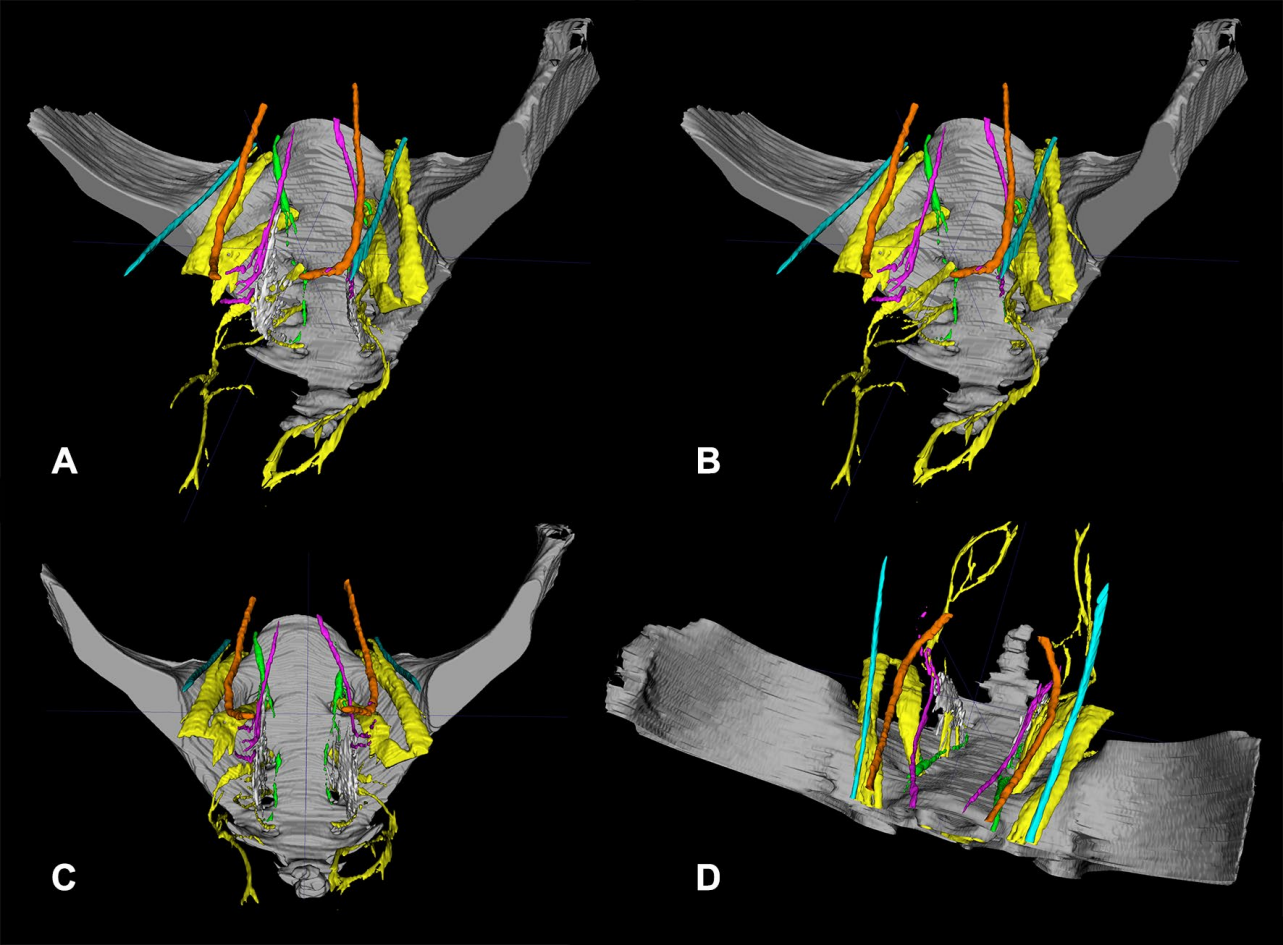
Different orientations showing the spatial relationship between the pelvic nerves in a male volunteer. **A** is an anteroposterior view slightly from the side. In **B**, the type of view is the same without the IHP (white) to show the PSNs, also known as the erigent pillar transferring parasympathetic fibers from the sacral nerves to the IHP. **C, D** illustrate a pure anteroposterior view such as encountered during, for example, a transanal total mesorectal excision and a view similar to what one would have during a laparoscopic total mesorectal excision, respectively. The following structures can be distinguished: ureter (orange), SNs/lumbosacral plexus/PN/LAN (yellow), ST (green), ON (turquoise), SHP/HN (pink), IHP (in **A**/**C**/**D**, white/distal side pink). (Color figure online)

**Fig. 3:**
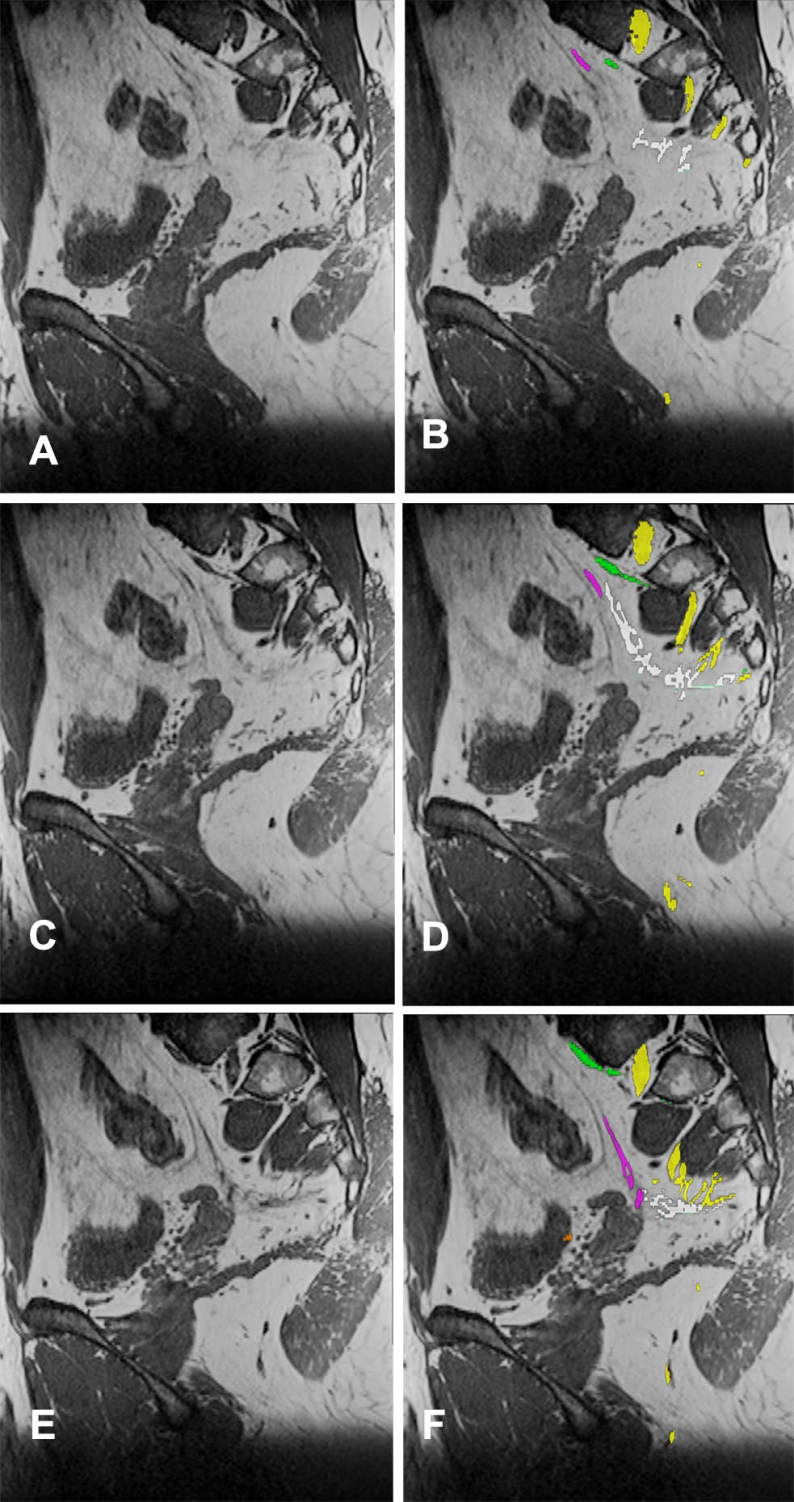
Three images in the sagittal orientation without (**A, C, E**) and with nerve segmentation (**B, D, F**) of the SNs (yellow), ST (green), HN (pink) joining the IHP (white). The infralevatory PN is delineated (yellow). The HN and IHP can best be discerned by sagittal navigation. (Color figure online)

The nerve identifiability and level of agreement are provided in Table 1. In 15 volunteers, the nerves were delineated with the T1 sequence. In four volunteers, the nerves were delineated with the PD sequence. In one volunteer, the nerves were delineated with the T2 sequence because of motion artifacts in the other sequences. Except for the levator ani nerve, the other nerves could be identified bilaterally in the vast majority of volunteers with a high level of agreement between the anatomist and the radiologist, except for the levator ani nerve (Table 1). Figure 1 and video 1 show a delineation of all nerves and their relationships with the pelvic organs in a male (Fig. 1A, video 1) and a female volunteer (Fig. 1B). In all volunteers, the lumbosacral plexus, SNs, and the ON could be fairly easily segmented (Fig. 2). The best way to identify the HN was through a medial-to-lateral navigation and vice versa in the sagittal plane (Fig. 3). In some cases, branches from the SNs, the so-called PSNs, could be distinguished, forming the IHP together with branches of the HN (Fig. 3). In some cases, the interconnecting fibers of the IHP, almost like a facial layer, could be distinguished as distinct structures. The connecting fibers between the ST and the IHP, the SSNs, could not be identified.

With respect to the quality score for the scan to recognize the HN (assessed by a Likert scale), no significant correlations were found between this score and BMI, age or gender. The Pearson χ^2^ *p* value for the comparison between gender and quality score was 0.329. A normal distribution was shown by means of a Shapiro–Wilk test for age (*p* = 0.120) and BMI (*p* = 0.424). The one-way analysis of variance (ANOVA) showed no differences for age in relation to the quality score (*p* = 0.170) and no differences for BMI in relation to the quality score (*p* = 0.265).

## Discussion

To our knowledge, this is the first study to report 3D MRI topography of all pelvic nerves at risk during pelvic visceral surgery in living humans. The likelihood of nerve delineation correctness was verified in three ways for validation purposes. Our study contributes to the earlier studies since these earlier studies analyzed only a part of the pelvic nerves and usually not in living humans. The LAN has neither been delineated on MRI in a living human nor on MRI in a human anatomic specimen.

Computed tomography (CT) imaging provides high resolution but with a poor contrast, and it is therefore not suitable to depict peripheral nerves. MRI is a noninvasive method which can provide high-resolution 3D images with a good contrast between anatomical structures. MR neurography is commonly performed in clinical routine to visualize nerve roots and large bundles of nerves, in order to identify points of compression or disruption. Most of the time, T2-weighted sequences with fat signal suppression are used [51, 52]. However, these sequences provide dark images with a contrast which is not ideal for the visualization of small pelvic nerves [53]. According to dedicated pelvic radiologists, high-resolution MRI is validated for the assessment of small pelvic nerves [53].

Another MRI acquisition method for the visualization of pelvic nerves is tractography. Diffusion tensor MR imaging (DTI) seems suitable for tractography of the lumbosacral plexus and sacral nerves [54]. In one study, tractography was successfully performed in the SNs in 10 healthy adults and in one 12-year-old patient [54]. However, the PN could not be delineated in the majority of cases. The authors concluded that DTI had its limitation and should not replace anatomical plexus imaging but it could offer valuable complementary information. The main reason for the failure of DTI to depict smaller nerves was that tractography of voxels on the basis of water molecules could be hampered by a low signal/noise ratio (SNR) of peripheral nerves [53], oversensitivity detecting all nerves fibers, also nonspecific [55], and crossing fibers resulting in false-positive or false-negative tractography [56]. In addition, tractography of the brain was processed via specific algorithms. Contrary to the brain, the pelvis is not a single organ but an inhomogeneous area made up of different tissues, complicating the use of these algorithms. Finally, DTI was also associated with a possible human error resulting in false-negative tractography since the most representative voxels necessitated a manual selection.

In 2014, Bertrand et al. performed pelvic 3-T MRI neurography and dissections in eight adult human anatomic specimen [7]. They attempted to identify the HN, IHP, PSN, and cavernous nerves, and compared several key points of this innervation and three anatomical references on MRI to the same key points and references after dissection. They tested several settings including T1, T2, T1 fat saturation, T2 fast relaxation fast spin-echo, and different diffusion settings with 1–2.5-mm slice thickness. They reported a congruence between these key reference points on MRI compared with the dissections, and they concluded that MRI was suitable to detect autonomous pelvic innervations [7]. However, they did not delineate the ST, PN, LAN, and ON.

In 2007, Mauroy et al. performed pelvic MRI and dissections of the IHP in 22 female human anatomic specimens to correlate anatomical data with MRI in order to determine the IHP path [57]. MRI with turbo spin-echo T2 sequences was performed in five female patients with a 1.5-T MRI scan in three planes with slice thickness of 3 mm without a T1 sequence. They reported a good anatomical radiological correlation using MRI for the IHP with simple points of reference.

Brown et al. performed T2-weighted MRI using a 1.5-T system in human anatomic specimen sections and in patients before they underwent TME. They compared anatomical dissections of sagittally sectioned hemi-pelvises with MRIs obtained in vivo and concluded that they could depict the IHP [51].

In our series, multiple sequences were scanned in different planes as mentioned in the protocol. The T1- and PD-weighted sequences provided images with a contrast which was better adapted to the visualization of small pelvic nerves compared with T2-weighted sequence. This coincided with the recent findings of pelvic radiologists performing MR neurography of the lumbosacral plexus and its pelvic neural branches [53]. In our experience, the visualization of pelvic nerves was best achieved in the axial plane, and special care was taken to optimize the resolution in this plane while accepting longer scanning times (up to 18 min) in the axial plane. The PD scan took longer than a T1 scan (18 vs. 12 min) did. Consequently, this sequence was more prone to motion artifacts.

The SHP, HN, and IHP could be bilaterally identified in 14 (70%), 16 (80%), and 14 (70%) of the 20 volunteers, respectively. In two of four cases (50%) in which no HN could be identified, there were at least two or more factors hindering nerve delineation, namely motion artifact (patient motion, bladder contractions, bowel contractions), inappropriate emptying of the bladder or fast filling and a low pelvic fat amount. In the other two scans, there was a motion artifact. In these cases, the signal intensity between nervous tissue and neighboring other structures was too little to visualize the HN and for accurate delineation. This coincided with a low mean quality score of 2 to recognize the HN on these scans (assessed by a Likert scale 0–5).

A limitation in finding and delineating nerves remained the resolution of the images. They were acquired with a 3-T MRI system, which offered a substantially higher SNR compared with a traditional 1.5-T MRI system. However, even with this higher magnetic field, the likelihood of missing small nerve branches with a width of less than 1 mm was significant, given the resolution chosen in this study (0.80 × 0.80 × 1.0 mm^3^). Therefore, it was not possible to recognize smaller, more distal nerve branches. Acquiring images with an even higher resolution was possible, but at the expense of a prolonged acquisition time and decreased SNR. We tried to find the best compromise by choosing an image resolution higher than the common resolutions used in MRI, but still compatible with time constraints of clinical routine. This resolution proved to be adapted to find most of the nerves of interest.

Characteristically, the bigger nerves (lumbosacral plexus, SNs, ON, and ST) could be distinguished on MRI by their fascicles. For the smaller nerves (SHP, HNs, IHP, PN, and LAN), a distinction could be made on the basis of a higher signal intensity than the intensity associated with other structures like the ureter and vessels, although the assessment of differences in signal intensity is made by visual inspection and is therefore somewhat subjective. The intensity of these nerves compared with the bigger nerves did not seem to differ much. The explanation for this finding might be that even the smallest pelvic nerves like the LAN are myelinated [45]. To our knowledge no data exist on inter-rater reliability for MRI pelvic nerve topography between radiologist, surgeon and anatomist.

Clinical routine pelvic MRI does not require the high level of detail for the depiction of peripheral nerves. At our hospital, these scans were acquired with a minimal slice thickness of 2 mm for the T2-weighted sequences and a 3.5 slice thickness for the T1-weighted sequences. For this reason, these scans were found to be unsuitable for topographic nerve mapping in a retrospective way. To summarize, T1-weighted high-resolution 3-T MRI seems to be the optimal sequence to depict normal anatomy of the peripheral pelvic nerves.

Although MRIs of anatomic specimens are not influenced by motion artifacts, they are hindered by cell death and temperature changes, rendering different contrasts compared with MRIs performed in vivo, and requiring adaptive setting [7]. The current study was performed in volunteers. For this reason, no contrast agents were used and nonenhanced images were acquired. Consequently, a noncontrast angiographic sequence was used including a low velocity encoding (1 cm/s), allowing for the visualization of large and smaller blood vessels, where blood velocity was reduced. This sequence was helpful to distinguish the PN and pudendal artery which were very close to each other. All in all, angiographic images were useful for 16 of the 19 volunteers in which this sequence was acquired. The next step to optimize and verify the reproducibility of this method of 3D nerve topography would logically be a clinical study with enhanced angiographic MRI. Common contrast agents in MRI such as gadolinium chelates could help to differentiate between vessels and nerves and thereby speed up the process. Anticholinergic agents might also be helpful by lowering bowel wall activity, thereby decreasing motion artifacts.

The ability to preoperatively identify the pelvic nerves on a medical imaging modality would help surgeons to better inform patients about expected functional results and to plan the operative strategy. These topographic maps are expected to be applicable during preoperative planning and intraoperative pelvic stereotactic navigation which is currently being developed for pelvic surgery [5]. Such an application during the laparoscopic stage of TME could facilitate dissection in the danger areas for nerve injury as previously described: the SHP at the level of the inferior mesenteric artery origin, the right and left HNs at the level of their origin, the IHP due to medial tenting as the rectum is retracted medially, and the IHP at the level of the seminal vesicles [58]. The first pilot cases of stereotactic navigation during transanal TME are already reported [4]. During a transanal approach, its application is supposed to prevent iatrogenic damage to the urethra and nerves and to help maintain the appropriate plane of dissection. A future application of individual 3D nerve maps in stereotactic navigation seems to be the logical next technological advancement to further improve the quality of care. To facilitate this, two projects are currently underway. Algorithms are currently developed to assist with semiautomatic nerve segmentation, thus facilitating the individualized preoperative mapping of the patient’s pelvic innervation. In addition, the positional changes and deformity of the pelvis are determined between scanning and different operating positions in order to facilitate the use of stereotactic navigation. The accuracy of neoadjuvant radiotherapy is also expected to improve by these developments facilitating image-guided radiotherapy when MRI is coupled to a Tele-radiotherapy unit [59]. Finally, a comparison between the preoperative and postoperative nerve statuses on MRI is valuable from a scientific perspective, providing insight into the influence of iatrogenic nerve damage on functional outcome. From a training perspective, potential benefits include a better understanding of the relative location of structures and a shorter learning curve.

In short, the current study shows that pelvic nerves at risk of damage during pelvic visceral surgery are visible on 3-T MRI. A specific knowledge of their course along anatomical landmarks and dedicated scanning protocols allows for 3D mapping. This opens up new opportunities such as its application in stereotactic navigation during pelvic visceral surgery.

## Electronic supplementary material

Below is the link to the electronic supplementary material.

Supplementary material 1 (M4V 21170 KB)

Supplementary material 2 (DOCX 42 KB)
