# Supplementary material for: A step towards stereotactic navigation during pelvic surgery: 3D nerve topography
Source: Surg Endosc. 2018 Feb 12;32(8):3582–91. doi: 10.1007/s00464-018-6086-3 (PMC6061054; doi:10.1007/s00464-018-6086-3)
Supplement: Supplementary file 2 — Supplementary material 2 (DOCX 42 KB) [file 464_2018_6086_MOESM2_ESM.docx]

**Supplementary Table 1:** Detailed search strategy.

| **Database** | **Time span** | **Search strategy** |
| --- | --- | --- |
| MEDLINE (Pubmed) | January 1947 to 27/2/17 | #1 non-MeSH term ‘superior hypogastric plexus’  #2 non-MeSH-terms ‘hypogastric nerve’  #3 MeSH-terms ‘hypogastric plexus’  #4 MeSH-terms ‘obturator nerve’  #5 MeSH-term ‘pudendal nerve’  #6 non-MeSH term ‘levator ani nerve’  #7 non-MeSH term ‘sympathetic trunk’ and MeSH-term ‘anatomy’  #8 non-MeSH term ‘sacral sympathetic trunk’ |
| EMBASE (OvidSP) | <1966, up to 27/2/17 | #1 superior AND hypogastric AND plexus  #2 hypogastric AND nerve AND anatomy  #3 hypogastric AND plexus AND anatomy  #4 obturator AND nerve AND anatomy  #5 pudendal AND nerve AND anatomy  #6 levator AND ani AND nerve AND anatomy  #7 sympathetic AND trunk AND anatomy |
| Science Citation Index Expanded (http://webofknowledge.com) | 1900 to 27/2/17 | #1 superior hypogastric plexus  #2 hypogastric nerve  #3 hypogastric plexus  #4 obturator nerve  #5 pudendal nerve AND anatomy  #6 levator ani nerve  #7 sympathetic trunk AND anatomy |

**Supplementary Figure:** Study flow diagram.

10 additional records identified through other sources

8168 records identified

through database searching

6192 records after duplicates removed

6192 records screened

6034 records excluded

97 full-text articles excluded, because: 95 did not report on the relevant nerve/ plexus and related landmark, 1 was a review study, 1 study of which anatomical specimens overlapped with another study that was included*

158 full-text articles assessed for eligibility

61 studies included in qualitative synthesis

61 studies included in quantitative synthesis

* Personal communication to author.

**Supplementary Text: MRI protocol.**

The subjects were examined with a 3-Tesla MR system (Verio, Siemens Healthcare, Erlangen, Germany). A 3D T1-weighted SPACE (Sampling Perfection with Application of optimized Contrasts using different flip-angle Evolutions) sequence was acquired with the following parameters: field of view (FOV)240*160mm², echo time (TE) 23ms, repetition time (TR) 400 to 600ms depending on the specific absorption rate (SAR) limitation, acceleration factor GRAPPA 2, 2 excitations, in plane resolution 0.80*0.80mm², 208 1mm-thick axial slices, acquisition time (TA) 10 to 15 minutes depending on the TR. A 3D T2-weighted SPACE sequence was acquired with the same parameters, except TE/TR=130/1600ms and TA=12min48. During the study, two adjustments were made to the protocol in order to optimize the nerve tracking capability. Non-enhanced angiographic images were acquired with a phase contrast sequence (same FOV, TE/TR=16.7/41.3ms, in plane resolution 1.3*1.0mm², 85 1.4mm-thick slices, velocity encoding 1cm/s, 3 excitations, TA=14 min). A 3D proton-density (PD)-weighted SPACE sequence was acquired with the parameters of the T1-weighted SPACE sequence, except TE/TR=22/1600ms and TA=17min52.

**Supplementary Table 2:** Studies identified for each nerve or plexus.

| **Nerve/ plexus** | **Studies included for qualitative synthesis** |
| --- | --- |
| Superior hypogastric plexus* | Lee 1973[28] |
|  | Havenga 1997[29] |
|  | Van Schaik 2001[30] |
|  | Mauroy 2003[31] |
|  | Bissett 2007[27] |
|  | Paraskevas 2008[32] |
|  | Lu 2009[33] |
|  | Shiozawa 2010[34] |
|  | He 2010[35] |
|  | Bertrand 2014[7] |
|  | Ripperda 2016[26] |
|  | Fermaut 2016[36] |
| Hypogastric nerve^†^ | Havenga 1996[29] |
|  | Mauroy 2003[31] |
|  | Hounnou 2003[37] |
|  | Mauroy 2007[38] |
|  | Spackman 2007[39] |
|  | Alsaid 2009[40] |
|  | He 2010[35] |
|  | Moszkowicz 2011[41] |
|  | Bertrand 2014[7] |
|  | Li 2015[42] |
|  | Ripperda 2016[26] |
|  | Fermaut 2016[36] |
| Inferior hypogastric plexus^‡^ | Baader 2003[43] |
|  | Mauroy 2003[31] |
|  | Akita 2003[47] |
|  | Hounnou 2003[37] |
|  | Ali 2004[44] |
|  | Spackman 2007[39] |
|  | Mauroy 2007[38] |
|  | Alsaid 2009[40] |
|  | He 2010[35] |
|  | Moszkowicz 2011[41] |
|  | Acar 2012[45] |
|  | Bertrand 2013[48] |
|  | Bertand 2014[7] |
|  | Li 2015[42] |
|  | Kraima 2016[46] |
|  | Ripperda 2016[26] |
|  | Fermaut 2016[36] |
| Pudendal nerve^§^ | Mahakkanukrauh 2005[52] |
|  | Lazarou 2008[54] |
|  | Grigorescu 2008[49] |
|  | Pirro 2009[51] |
|  | Colebunders 2011[53] |
|  | Gabrielli 2011[50] |
| Levator ani nerve^#^ | Borirakchanyavat 1997[55] |
|  | Barber 2002[23] |
|  | Wallner 2006[56] |
|  | Wallner 2008[57] |
|  | Grigorescu 2008[18] |
|  | Lazarou 2008[54] |
|  | Nyangoh Timoh 2016[58] |
|  | Loukas 2016[59] |
| Obturator nerve^¶^ | Kendir 2008[21] |
|  | Won 2016[22] |
| Sympathetic trunk** | Pick 1957[24] |
|  | Barber 2002[23] |
|  | Guvencer 2009[25] |
|  | Ripperda 2016[26] |

* Of these twelve studies, 10 studies[28-36] report a division of the SHP at the level or just caudal to the level of the sacral promontory. These studies include dissections of in total 79 male and 37 female cadavers. Two studies[7, 27] do not report the level of division, but such a division is confirmed by pictures of the cadaver dissection.

† Of the twelve included studies, nine studies[29, 31, 35-41] report a course medial to the internal iliac vessels (hypogastric vessels) comprising 18 male adult, 38 female adult cadavers, 2 male fetuses and 6 female fetuses. The remaining three studies do not report the type of course in relation to the internal iliac vessels, but does show a course medial to these vessels in the dissection pictures[7, 26, 42]. Of two studies in which female cadaveric hemipelvises[17, 38] were dissected, one study[17] was excluded because some of the specimens were included in both studies (personal communication with main author).

‡ Of the 17 included studies, thirteen studies [26, 31, 36-46] (comprising 77 male adult, 107 female adult and 2 male and 16 female fetuses) report as a main positional reference the terminal ureter which crosses the IHP cranially for the greater part. In some cases a few IHP fibers are described to cross cranial to the ureter. The remaining four studies[7, 35, 47, 48] do not report the type of course in relation to the ureter, but such a course is confirmed by pictures of the cadaver dissection.

§ Of 29 studies of which the full text was assessed only 6 reported information with respect to the relation of the PN to the pudendal vein/ artery. Of these studies, three studies[49, 50, 54] comprising 47 adult cadavers of both sexes reported a course of the PN medial to the pudendal artery when exiting the pelvis through the greater sciatic foramen. Another study[51] comprising 11 male and 9 female cadaveric dissections, reported such a course of the PN in 80% of cases. The remaining two other studies[52, 53] did not report the type of course in relation to the pudendal vessels, but such a course is confirmed by pictures of the cadaver dissection.

# In total eight studies comprising 159 female adult cadaveric dissections and sections of 4 male and 12 female fetuses, assessed the presence and course of the LAN[18, 23, 54-59]. For the LAN, they reported a supralevatory course parallel to the PN. The biggest study comprising 100 cadaveric dissections reported the presence of the LAN in all cases[59].

¶ Only two studies comprising 67 male and 28 female cadaveric dissections, show the relation of the proximal ON to the pelvic vessels by means of pictures of the dissections and schematic drawings of anatomical relations[21, 22]. All these illustrations show a course of the proximal ON dorsal to the confluens of the internal and external iliac vein.

** Three studies comprising dissections of the ST in 15 male and 12 female cadavers, report a presacral course medial from the sacral foramina[23-25]. A fourth study comprising 17 male cadavers reports a course medial or just anterior to the sacral foramina[26].
